# Direct and indirect damage zone of radiofrequency ablation in porcine lumbar vertebra

**DOI:** 10.3389/fonc.2023.1138837

**Published:** 2023-02-22

**Authors:** Chao Zhang, Jinyan Feng, Yongheng Liu, Yan Zhang, Weijie Song, Yulin Ma, Xiuxin Han, Guowen Wang

**Affiliations:** ^1^ Department of Bone and Soft Tissue Tumor, Tianjin Medical University Cancer Institute and Hospital, National Clinical Research Center for Cancer, Key Laboratory of Cancer Prevention and Therapy, Tianjin’s Clinical Research Center for Cancer, Tianjin Medical University Cancer Institute and Hospital, Tianjin, China; ^2^ Department of Animal Laboratory, Tianjin Medical University Cancer Institute and Hospital, National Clinical Research Center for Cancer, Key Laboratory of Cancer Prevention and Therapy, Tianjin’s Clinical Research Center for Cancer, Tianjin Medical University Cancer Institute and Hospital, Tianjin, China

**Keywords:** radiofrequency ablation, vertebrae, tumor model, direct damage, indirect damage

## Abstract

**Objectives:**

To explore the direct and indirect heat damage zone of radiofrequency ablation (RFA) in porcine vertebrae and to verify the safety of RFA in a vascularized vertebral tumor model.

**Methods:**

RFA was performed in the porcine lumbar vertebrae. Magnetic resonance (MR) imaging, hematoxylin and eosin (HE), and terminal deoxynucleotidyl transferase dUTP nick end labelling (TUNEL) were used to assess the extent of direct and indirect injuries after RFA. The cavity of lumbar vertebrae was made, and the adjacent muscle flap was used to fill the cavity to make a vertebrae tumor model. RFA was performed in the vascularized vertebral tumor model.

**Results:**

T1-weighted images showed a hypointensive region in the center surrounded by a more hypointensive rim on day 0 and 14. T2-weighted images showed that RFA zone was hypointensive on day 0. On day 7, hypointensity was detected in the center surrounded by a hyperintensive rim. HE showed that the RFA zone could be clearly observed on day 14. Thin bone marrow loss areas were seen around the RFA zone, which was consistent with the hyperintensive rim on the T2-weighted images. TUNEL showed a large number of apoptotic cells in the RFA zone. During RFA in the vertebral tumor model, the temperature of all monitoring positions was less than 45 °C.

**Conclusion:**

Using *in vivo* experiments, the effective zone of RFA was evaluated by MR imaging and pathology, and the direct and indirect damage range were obtained. The safety of RFA was verified by RFA in a vascularized vertebral tumor model.

## Introduction

Bone is the third most common metastasis site in all cancer patients, and the spine is the most common metastasis site. Of patients with a malignant tumor, 10-40% will eventually have spinal metastasis (1–3). Spinal metastasis often leads to neurological dysfunction, sphincter dysfunction, hypercalcemia, pathological fracture, and even paralysis (4). In addition to systemic treatment, the main purpose of spinal metastases treatment is to minimize pain, maintain mechanical stability, and improve the quality of life. Although the main method for the treatment of painful bone metastasis is radiotherapy, the pain relief after radiotherapy may be partial, delayed, and temporary (5). Pain caused by spinal metastases is usually not effectively alleviated by systemic therapy such as chemotherapy, hormone therapy, radiotherapy, and bisphosphonates (6). In the past decade, RFA has developed rapidly in the treatment of spinal metastases, and its efficacy has been recognized (7–9).

RFA has been widely used as a minimally invasive treatment in bone metastases, especially in palliative pain treatment of the spine, pelvis, long bone, etc. (10, 11). The molecular oscillation of charged tissue in the RFA zone produces friction heat, which leads to coagulation necrosis of the tumor (11–13). When the temperature of RFA reaches 45°C, irreversible necrosis can occur within a few hours. When the temperature reaches 50-55°C, irreversible cell damage can occur within 4-6 minutes. When the temperature reaches 60-100°C, protein coagulation necrosis occurs immediately. When the temperature reaches 100-110°C, the tissue will be carbonized and vaporized (14).

RFA can effectively kill tumor cells without damaging the stability of the vertebra, thereby reducing the risk of pathological fracture. Several studies have demonstrated that percutaneous RFA is a safe technique that can be very effective in relieving pain in patients with spinal metastases (15–18). RFA has been widely used in palliative treatment of spinal metastases, but there are corresponding side effects of RFA, including bleeding, infection, skin injury, organ injury, spinal cord, and nerve root injury (19, 20). Several reports evaluated the RFA damage lesion by MR imaging and HE, and evaluated the safety of RFA, which has certain guiding significance for the clinical application of RFA (21, 22). RFA in the treatment of liver tumors, heat injury includes two stages, direct heat injury and indirect heat injury (23). But there was no study to further clarify the indirect heat injury of RFA in vertebral metastases.

Therefore, we need to study the direct and indirect heat damage zone of RFA in the vertebra. In this study, the damage zone of RFA in porcine lumbar vertebrae was studied *in vivo*. The area of coagulation necrosis was evaluated by MR imaging and HE. TUNEL was used to evaluate the apoptosis of vertebral cells. The tumor model of vertebra was constructed to verify the safety of RFA.

## Materials and methods

Institutional animal care ethics approval was obtained for the study. Eight Bama miniature pigs, weighing 35-40 kg, were used in this study; six pigs were used for RFA in normal vertebrae, and two pigs were used for RFA in tumor model vertebrae. Tarlov score was used to evaluate the walking function of pigs before and after RFA (24). Tarlov score can be divided into six grades. Grade 0: no activity of hind limbs, no weight bearing. Grade 1: the hind limbs can move occasionally but cannot bear weight. Grade 2: the hind limbs move frequently or forcefully but cannot bear weight. Level 3: the hind limbs can support the body weight and walk 1-2 steps. Grade 4: walkable with only mild impairment. Grade 5: normal walking.

### Radiofrequency ablation system

RITA ^®^ The 1500x generator (angiodynamics, Inc., Manchester, Ga., USA) can emit 460khz RF current with a maximum power of 250W. The perfusion pump can continuously infuse normal saline into the probe to increase the range of RFA. Probe(17g): starburst flex (uniblade) unipolar perfusion probe. Continuous saline infusion at the tip of the probe can effectively increase the RFA zone, which has been approved by FDA for the treatment of bone tumors.

### Anesthesia

To begin, sedative drugs were delivered through an intramuscular injection of Sumianxin (0.2ml/kg) and 3% phenobarbital (1ml/kg). It took about 5-10 minutes for the pig to fall asleep. The vein channel was established through the ear vein, and the intravenous indwelling needle was inserted. 8-10ml of general anesthesia drug was injected (propofol injection, 2mg/kg, Fentanyl citrate injection, 2ug/kg, Rocuronium injection, 1mg/kg). Endotracheal intubation was performed with an insertion depth of about 28cm. After successful intubation, a ventilator was connected, and anesthetics were given continuously. The vital signs such as respiration, heart rate, and electrocardiogram were observed.

### Surgery

The Bama miniature pig was fixed on the operating table in a prone position. The hair of the waist, bilateral thighs, and buttocks were shaved. Two negative plates were applied to the buttocks and thighs of the pig. The surgical zone was disinfected with Iodophor, and sterile operating sheets were laid down. The posterior median incision of the lumbar spine was made with a length of about 20cm. The skin, subcutaneous, and fascia were incised to separate the erector spinae muscle and expose the spinous process, lamina, articular process, and transverse process of l1-l6. Through the right pedicle approach, the RFA channel was made. The needle entry point was the intersection of the superior articular process and transverse process, about the middle line of transverse process, and the inclination angle was approximately 25 to 30 degrees.

### RFA in normal vertebrae

Previous literature reported that the number of lumbar vertebrae in pigs was 6-7 (25). The experimental animal in this study was the Bama miniature pig, and each pig had six lumbar vertebrae. RFA was performed in L1, L2, L3, L4, and L5 vertebrae of six Bama miniature pigs, and L5 vertebra was not ablated as control group. The ablation parameters were set as power 35W, temperature 70 °C, active tip 1cm, and ablation time 20 minutes. Thermocouples were placed in the spinal canal, the pedicle hole, and the anterior edge of the vertebra to monitor the temperature in real time. MR imaging (GE, 3.0T discovery, MR750) was performed on 0, 7, and 14 days after RFA. The scanning sequences were T1-weighted and T2-weighted. In T1-weighted and T2-weighted images, the longest diameter of RFA was measured.

The three groups of pigs were euthanized at three separate time points, and then the lumbar vertebrae were taken out. A high-precision hard tissue slicer was used to cut the vertebrae to obtain a complete cross-section of the vertebral body. The thickness of the section was about 2 mm. The maximum diameter of ablation range of gross specimens was measured. Then the special embedding box was used for embedding, ethylene diamine tetraacetic acid (EDTA) was used for decalcification, and the decalcification of samples was observed regularly. Finally, HE and TUNEL were used to evaluate the range of RFA. According to the effective range of HE staining, the maximum diameter of RFA was measured.

### RFA in vertebral tumor model

Two Bama miniature pigs were used to make vertebral tumor models. First, an electric grinding drill was used to grind along L1, L3, and L5 pedicle direction, and a quasi-circular cavity with a diameter of about 1.7cm was ground in the upper 1/3 of the vertebra to ensure the integrity of the surrounding bone. The adjacent erector spinae muscle was separated to form the adjacent muscle flap with blood supply, which was filled in the vertebra to construct the vertebral tumor model. RFA was performed on the vertebral tumor model. The ablation parameters were set as power 35W, temperature 70°C, needle length 1cm, and ablation time 20 minutes. Temperature measurement points were arranged in the spinal canal (posterior cortex of vertebra near spinal cord), nerve root foramen, and anterior edge of vertebral body, and thermocouples were used to monitor the temperature in real time.

### Statistical analysis

IBM SPSS 23.0 statistics software was used for statistical analysis. The maximum diameters of the RFA zone in MR imaging, HE, and gross samples were measured. ANOVA was used for comparison between the three groups, α=0.05 was taken as the test level, and P < 0.05 showed a statistical difference.

## Results

### Neurological function

In this study, RFA was performed on the lumbar vertebrae of eight Bama miniature pigs. All Bama miniature pigs were evaluated with Tarlov score before and after the experiment to evaluate the walking function of lower limbs and sphincter function. All pigs could walk normally after RFA. Tarlov score was 5. No pigs had sphincter dysfunction.

### Gross specimen

**Figure 1:**
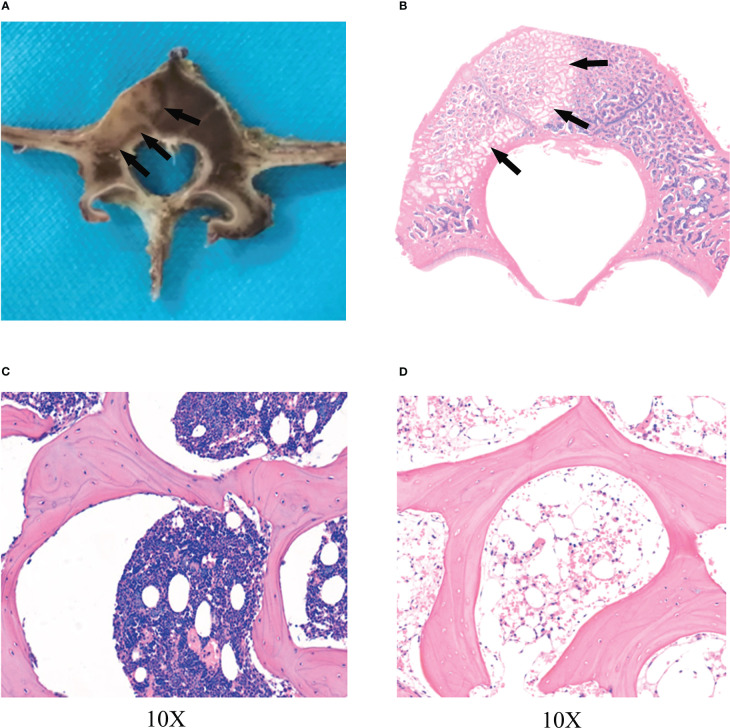
The zone of RFA. **(A)** Radiofrequency ablation (RFA) lesion in vertebra by transverse plane. The zone of RFA in the vertebra showed gray-white changes, with thin layers of gray-black rim (black arrows) around the center on day 14. **(B)** Hematoxylin and eosin (HE) showed a clear radiofrequency ablation zone(black arrows) on day 14. A circular bone marrow deletion zone can be seen around radiofrequency ablation lesion. The trabeculae remained intact, but the number of bone marrow cells in the trabeculae and the number of osteoblasts covering the trabeculae were significantly reduced, especially in the surrounding zones (black arrows) of radiofrequency ablation. **(C)** The zone of radiofrequency ablation was observed at 10X. **(D)** Normal vertebra.

On day 0 after RFA, the transverse plane of the vertebrae showed no significant changes in the RFA zone compared to the normal vertebrae(**Figure 1**). On day 14 after RFA, the zone of RFA in the vertebra showed gray-white changes, with thin layers of gray-black rim around the center (**Figures 1A, B**).

### MR imaging

**Figure 2:**
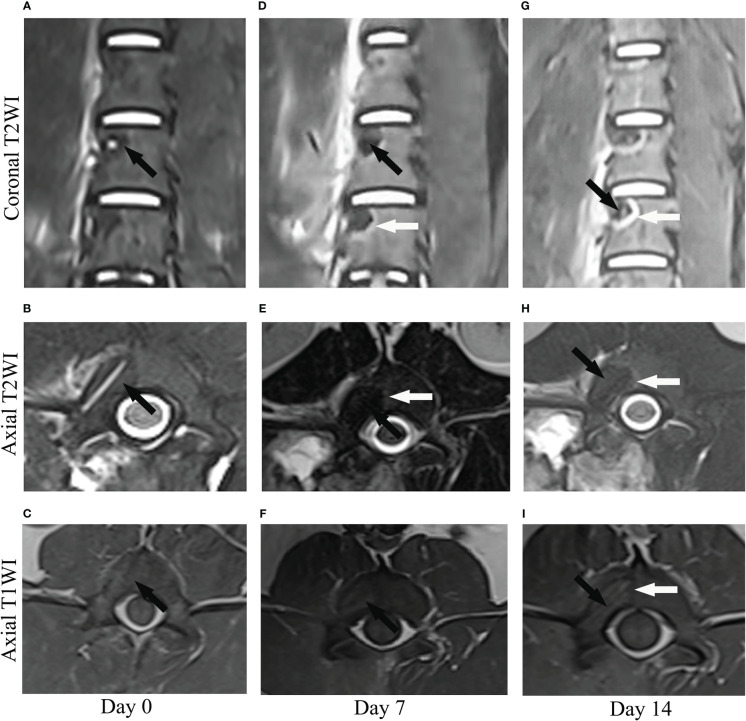
The image manifestation of a lesion on coronal and axial T2-weighted images (T2WI) and axial T1-weighted images (T1WI) at different time points (day 0, 7, 14) after radiofrequency ablation. **(A–C)** On day 0, the lesion showed hypointensity (black arrow) on coronal and axial T2WI and isointensity (black arrow) on axial T1WI. **(D–F)** On day 7, the lesion demonstrated hypointensity (Black arrow) at the center with hyperintensity (white arrow) at the periphery on coronal and axial T2WI and heterogenous intensity at the center with slightly hypointensity at the periphery on axial T1WI (Black arrow). **(G–I)** On day 14, the peripheral ring (white arrow) which manifested as hyperintensity on coronal and axial T2WI and hypointensity at the center was clearer than that of day 7.

The RFA regions were evaluated by coronal plane and axial plane. T1-weighted images (**Figures 2C, I**) showed hypointensity in the center surrounded by a more hypointensive rim on day 0 and 14. T2-weighted images showed the RFA zone was hypointensive on day 0 (**Figures 2A, B**). On day 7, the lesion demonstrated hypointensity at the center with hyperintensity at the periphery on coronal and axial T2WI (**Figures 2D, E**). On day 14, the hyperintensive rim was more obvious(**Figures 2G, H**), which was consistent with the hypointensive rim in T1-weighted images(**Figure 2I**). Compared with T1-weighted, T2-weighted images more clearly show the zone of RFA.

### HE

**Figure 3:**
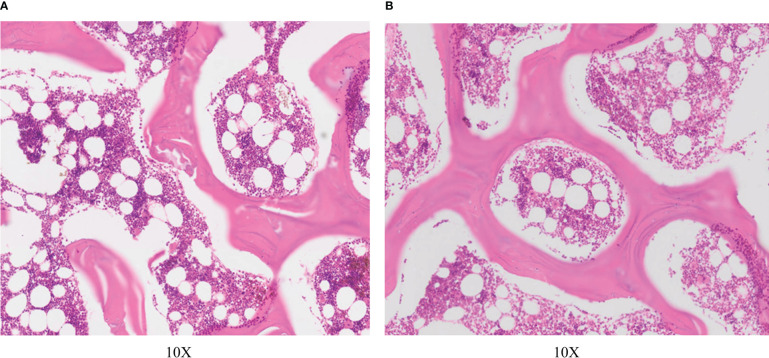
HE on day 0. **(A)** Normal vertebra. **(B)** Radiofrequency ablation zone.

On day 0 after RFA, HE showed no significant differences in trabecular structure, osteoblasts, and marrow composition between the RFA zone and the normal vertebra (**Figure 3**). On the 14th day after RFA, HE showed that the trabecular bone remained intact in the RFA zone. However, compared with normal vertebra, the bone marrow in the trabecula was significantly reduced, and the number of osteoblasts covered by trabecula in the ablation zone was also significantly reduced, especially in the peripheral zone of RFA. It formed a circular bone marrow loss zone (**Figures 1A, B**), which was consistent with the hyperintensive rim in MR images.

### TUNEL

**Figure 4:**
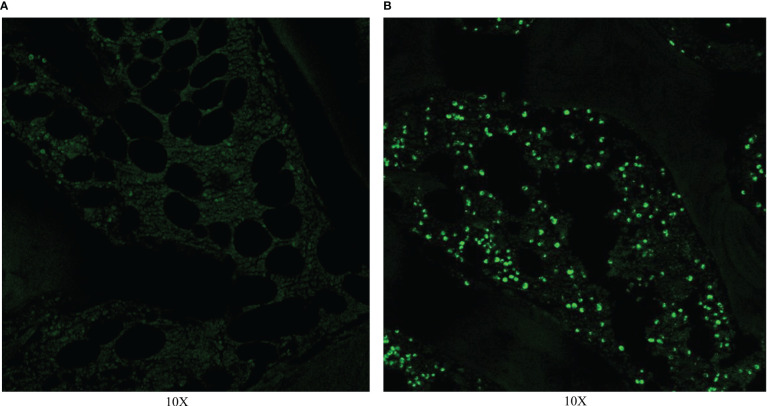
TUNEL on day 0. **(A)**TUNEL showed no apoptotic cell in normal vertebra. **(B)** TUNEL showed a large number of apoptotic cells in the ablation zone.

TUNEL was performed on 0 and 14 days after RFA. On day 0, TUNEL showed that a large number of apoptotic cells existed and the nuclei of apoptotic cells were green. Apoptotic cells were mainly distributed around the ablation zone, and were mainly bone marrow tissue (**Figure 4**).

### RFA diameter measurement

**Figure 5:**
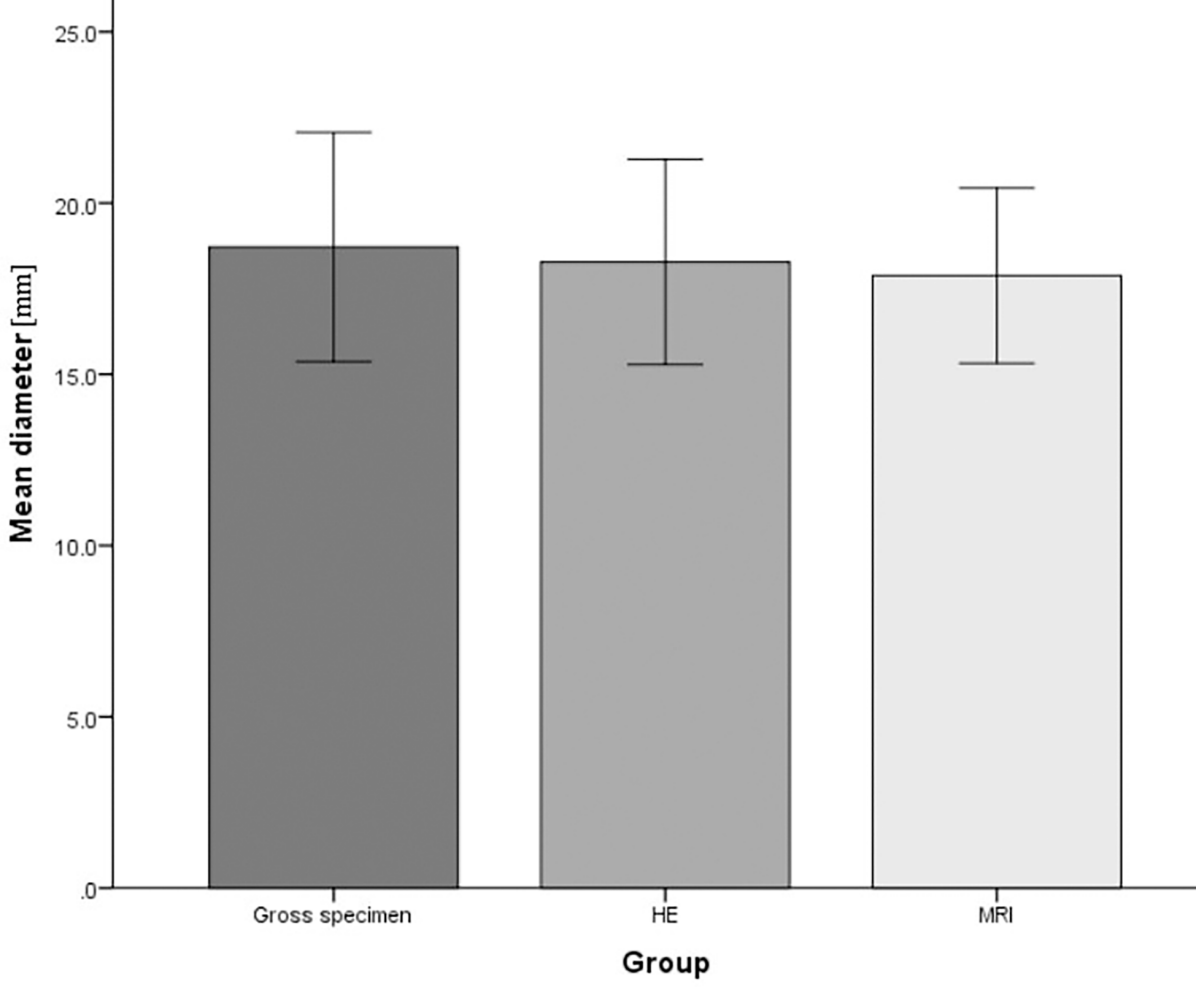
The diameter of radiofrequency ablation (RFA) in gross specimens, Hematoxylin and eosin (HE), and magnetic resonance (MR) imaging. The average diameter was 18.72 ± 2.69cm in gross specimens, 18.28 ± 2.41in HE, 17.88 ± 2.06cm in MR images.

The diameter of RFA was measured in gross specimens, HE, and MR images. The average diameter in gross specimens was 18.72 ± 2.69cm, that in HE was 18.28 ± 2.41, and that in MR images was 17.88 ± 2.06cm. The three measurement methods were not statistically significant(P=0.86) (**Figure 5**). The three measurement methods can be used as an effective method to measure the range of RFA.

### Safety in vertebral tumor model

**Figure 6:**
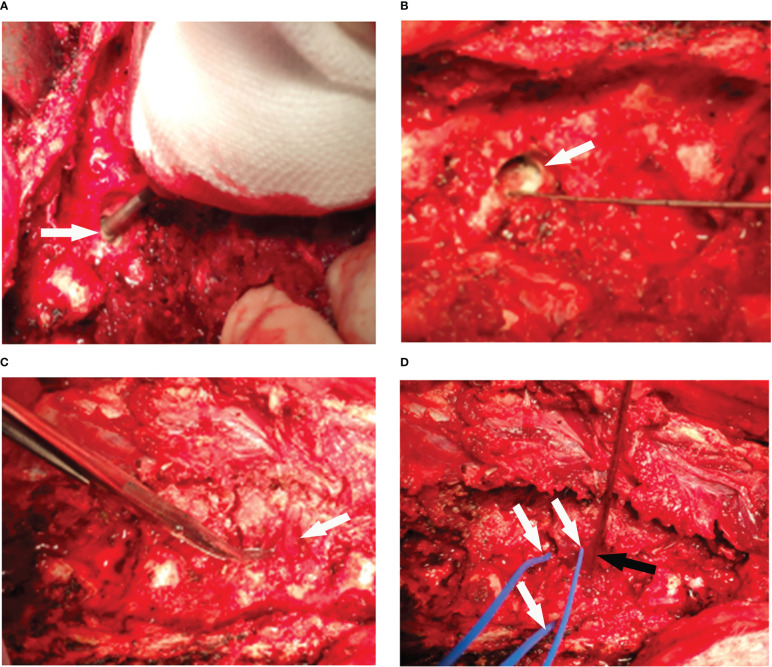
The process of making a vertebral tumor model. **(A)** The holes were drilled through the pedicle of vertebral with a grinding drill (arrow). **(B)** The cavity was made in vertebra (arrow). **(C)** The adjacent muscle flap was separated (arrow), and the vascularized muscle flap was used to fill the vertebral cavity. **(D)** Radiofrequency ablation was performed in the vertebral tumor model (black arrow), and the temperature of the peripheral spinal cord, nerve root foramen, and anterior edge of vertebra were monitored (arrows) in real time.

**Figure 7:**
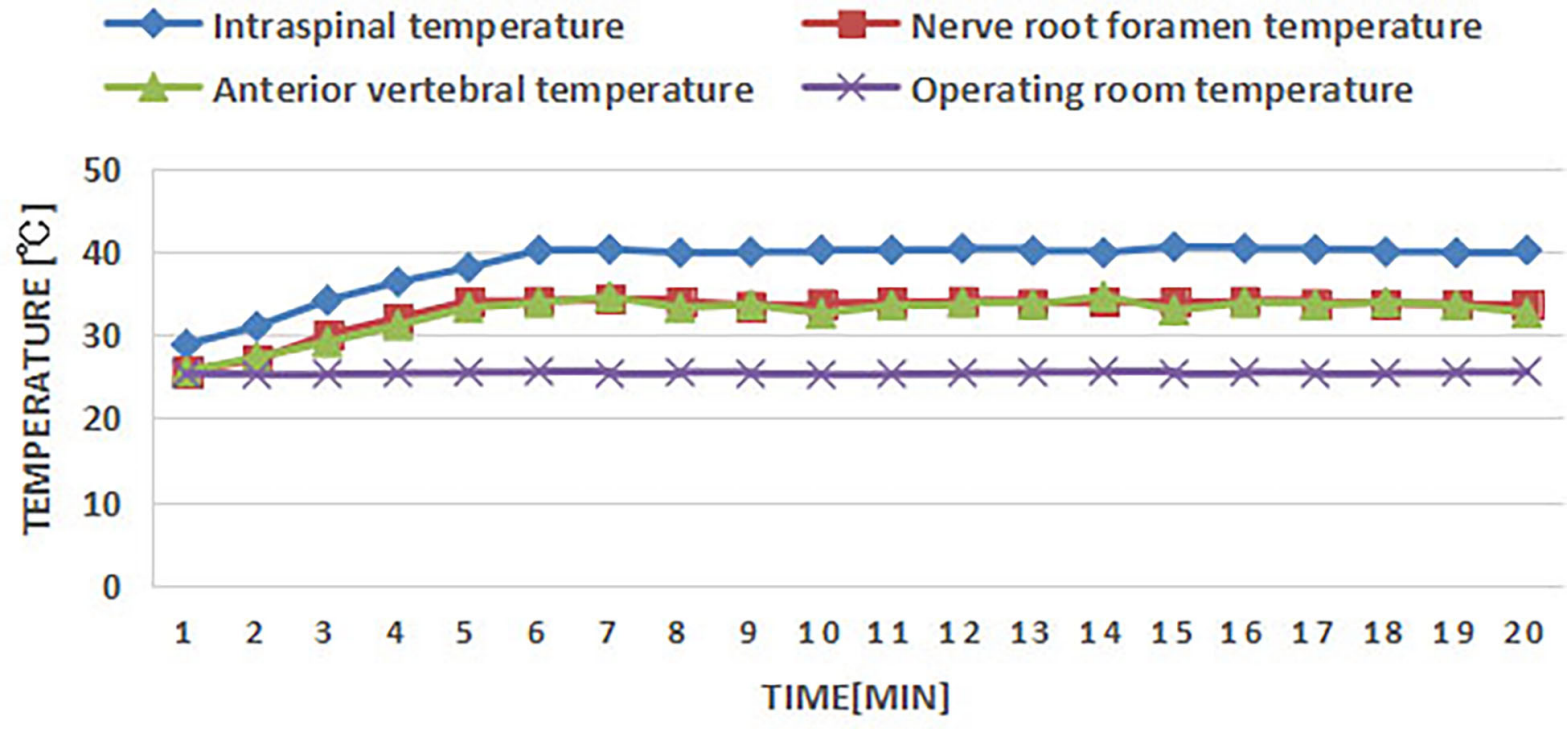
Temperature measurement points in spinal canal, nerve root foramen, and anterior edge of vertebrae.

According to the previous study, muscle was used to fill the vertebrae to make the vertebral body tumor model (26). In this study, a grinding drill was applied to the L1, L3, and L5 vertebrae through the right pedicle to make the cavity (**Figures 6A, B**), and the adjacent erector spinae muscle flap was separated, and the vascularized muscle flap was filled into the vertebrae cavity to make the vertebral body tumor model (**Figure 6C**). RFA was performed in the tumor model to monitor the temperature in the spinal canal, nerve root foramen, and anterior edge of vertebrae (**Figure 6D**). The temperature of all monitoring points was within the safe range (**Figure 7**). The safety of RFA in a vertebral tumor model was verified.

## Discussion

RFA has been widely used in the clinical treatment of spinal metastases, but the related complications during RFA cannot be ignored. At present, the research on the distribution of a thermal field of RFA in vertebra is very limited. In this study, it was found that the RFA in pig vertebra was safe, and the effective therapeutic range could be obtained by controlling the parameters of RFA, including the action temperature, power, action time, and length of active tip. MR imaging evaluated the zone of RFA in vertebrae. The area of coagulation necrosis (14 days after RFA) can be observed by HE under the microscope. The area of apoptosis in the vertebrae can be observed by TUNEL. The direct and indirect damage range were obtained by MR imaging and HE.

You et al. (27) found that the local temperature around the RFA zone is inversely proportional to the distance when RFA is performed in the vertebra *in vitro*. In general, RFA equipment, relevant parameters, type of electrode, and conductivity of tumor tissue are the factors affecting the size of thermal lesions. The RFA electrode used in this study is a unipolar perfusion electrode, which is approved by the FDA and can be used in the treatment of bone tumors. Continuous drip of normal saline during RFA can increase the conductivity of the tissue around the electrode. Through continuous perfusion of normal saline, heat is dissipated to the tissue far from the electrode to enhance thermal conductivity. Therefore, continuous perfusion during RF ablation can enhance the ablation effect.

You et al. (27) found that there was a significant positive correlation between ablation zone and ablation time within a certain time, meaning a sufficient volume of RF ablation zone can be produced by adjusting the length of ablation time. In this study, the active tip was set at 1cm, the ablation temperature was set at 70°C, and the action time was set at 20min. The radiofrequency ablation in the vertebra was enough to ensure that the spinal cord, nerve root, and other important tissues would not suffer thermal damage, and the ablation range could be clearly observed through MR imaging.

The zone of ablation was evaluated by means of magnetic resonance imaging, gross specimens, and histopathological sections. The size by three means were consistent (28). In this study, the maximum diameter of pathology can be obtained by thin-layer sections of gross specimens. However, MR imaging has layer thickness. The layer thickness of MR images in this study was 3mm, which made it difficult to ensure that the maximum diameter can be obtained by tomography. Therefore, there will be an error between MR images and the maximum diameter of pathological sections, but this error is not statistically significant through statistical analysis in this study.

In an *in vivo* experiment, Pezeshki et al. (21) performed RFA of porcine vertebra, monitored the temperature around the vertebral body during RFA, and evaluated the neurological function after treatment, proving that RFA was safe. MR imaging assessments were conducted pre- and posttreatment. RF lesions were apparent in the T2-weighted sequences, which showed a combination of hypointensive and hyperintensive regions, often demonstrating a hyperintensive peripheral rim on day 14. In this study, RFA was performed on porcine lumbar vertebrae. The effective range of RFA can be clearly observed on days 0, 7, and 14 in T2-weighted images. The range of RFA can be more clearly observed on day 14, and there is a hyperintensive rim, which is consistent with the results of previous literature (21).

Studies have shown that after RFA, in addition to direct damage, the tissue will still suffer indirect damage after ablation is stopped (23). Clinical and experimental data showed that the tissue damage will continue after thermal ablation is stopped (23, 29, 30), but the mechanism of such damage remains to be studied. Related studies have reported that after hyperthermia, RFA should be delayed by 5-7 days to clearly observe the thermal lesion (23). The indirect injury mechanism may also be related to many factors, including cell apoptosis, macrophages, cytokine release, and ischemia-reperfusion injury. The related mechanism of indirect injury in vertebra needs to be further studied (23). In this study, it was found in MR imaging that a hyperintensive rim was observed around coagulation necrosis on the 7th and 14th days after RFA, and was clearer on the 14th day. At the same time, the scope of coagulative necrosis could be clearly observed by HE on 14th day, and a thin layer of bone marrow loss zone could be observed around the outermost periphery of coagulative necrosis, which was close to the hyperintensive rim of MR images. This study considered that this zone may be the indirect injury zone of RFA in the vertebra.

Spinal metastases are divided into osteogenic type, osteolytic type, and mixed type. Different types of bone destruction of spinal metastases have different electrical conductivity in the vertebra. According to previous studies, the temperature field distribution of RFA in vertebrae, and many studies were carried out in normal vertebral bodies (21, 27). Compared with spinal metastases, RFA in normal vertebral bodies has a large error in evaluating the temperature field area and safety. Relevant studies have reported that different vertebral metastasis models were made for RFA research, including filling muscle tissue to simulate metastasis (26, 31). But the tumor is characterized by rich blood supply, so filling muscle tissue to build a tumor model lacks this important feature of tumors. In this study, the vertebral cavity was made, and the adjacent vascularized muscle flap was separated to fill the cavity to simulate the vertebral tumor. Compared with previous studies, this model had blood supply, and the safety of RFA was verified in this vertebral tumor model.

The experimental animal of this study was pig. Compared with human vertebrae, the vertebrae of pig are smaller. The RFA power and temperature setting explored in this experiment were safe, but the relevant parameters need to be reset for RFA in human vertebral metastases. The experimental results can only be used as a reference. The action time in this study was 20 minutes. The zone of RFA can be better evaluated by MR imaging, but the optimal action time needs more research and further demonstration. Because it was difficult to make a spinal metastasis model in large animals, the zone of RFA in this study was carried out in normal vertebrae. The actual ablation range in vertebral tumors may change due to different tumor tissues.

## Conclusion

In this study, RFA was performed on Bama miniature pigs. Thermocouples were used to monitor the temperature in the spinal canal, nerve root foramen, and anterior edge of the vertebral body in real time to verify the safety of RFA. MR imaging and HE methods were used to evaluate the zone of direct and indirect damage of RFA. Whether indirect injury can cause thermal injury to the spinal cord and its mechanism need to be further studied. The model of lumbar vertebral tumor was made and RFA of vertebral tumor was performed to verify the safety, which provides a certain theoretical basis for the safe and effective development of perfusion electrode monopole needle in spinal metastasis.

## Data availability statement

The original contributions presented in the study are included in the article/supplementary material. Further inquiries can be directed to the corresponding authors.

## Ethics statement

The animal study was reviewed and approved by Tianjin Medical University Cancer Institute and Hospital. Written informed consent was obtained from the owners for the participation of their animals in this study.

## Author contributions

All authors listed have made a substantial, direct, and intellectual contribution to the work and approved it for publication.
